# Antibiotic prophylaxis and surgical site infections in orthognathic surgery – a retrospective analysis

**DOI:** 10.1186/s12903-023-03391-3

**Published:** 2023-09-25

**Authors:** Andreas Naros, Carola Helene Naros, Daniel Awad, Michael Krimmel, Susanne Kluba

**Affiliations:** Department of Oral and Maxillofacial Surgery, Tuebingen University Hospital, Osianderstrasse 2-8, Tuebingen, 72076 Germany

**Keywords:** Orthognathic surgery, Surgical site infection, Antibiotics

## Abstract

**Background:**

This study was conducted to determine surgical site infection (SSI) rates and potential risk factors as well as to evaluate antibiotic prophylaxis in orthognathic surgery.

**Methods:**

This retrospective observational study included patients who received orthognathic surgery. SSIs and their management were assessed for up to one year post-operatively. The applied antibiotic regime and other possible influencing factors (smoking, age, site of infection, drainage, duration of surgery, displacement distances, craniofacial malformations) were assessed.

**Results:**

In total 291 patient met the inclusion criteria (56.7% female). The mean age at surgery was 25.5 ± 8.5 years. Fifty-four patients (18.6%) were diagnosed with a craniofacial malformation. Relevant previous surgeries were documented in about one quarter of included patients (*n* = 75). Ninety-two percent of patients (*n* = 267) received intraoperative single-dose antibiotic prophylaxis. Surgical site infections occurred in 12.4% (*n* = 36) of patients. There was a significant association between postoperative infections and type of surgery (*P* = .037) as well as type of drainage (*P* = .002). Statistical analyses also revealed a higher prevalence of smokers (*P* = .036) and previous surgically assisted rapid palatal expansion (SARPE) (*P* = .018) in the infection group. Furthermore, no significant relationships were observed between postoperative infections and various co-factors (i.e. antibiotic regime, age at surgery, gender, associated craniofacial malformations, surgery duration, displacement distances, mandibular setback vs. advancement).

**Conclusion:**

Low rates of SSIs occurred following an intraoperative single-dose antibiotic regime. None of the SSIs had a significant effect on the final surgical outcome. Present data do not warrant escalation of the antibiotic regimen. Postoperative smoking and capillary drainage should be avoided.

## Background

Orthognathic surgery is a frequently performed elective maxillofacial procedure to correct skeletal dentofacial anomalies. The intervention is classified as a clean-contaminated class II-surgery [1] and surgical site infections (SSI) are a relevant problem. The data on infection rates vary enormously, from 1.4% to 33.4% [2, 3]. SSIs endanger the surgical outcome and represent a significant additional burden for the patient and the health care provider. Therefore, reducing the risk of infection is a high priority in treatment and has always been the subject of much scientific research. Various prophylactic measures have been established, among which antibiotic prophylaxis is discussed particularly frequent and controversially. The alarming facts from the WHO [4] on the development of resistance and the associated predictions on mortality underline the need to critically evaluate any application. To date, the antibiotic prophylaxis regimes applied have been very heterogeneous, ranging from single-dose pre-/intraoperatively [5, 6] to perioperative short-term application [7, 8] and administration over several days [9, 10]. The scientific results are very heterogeneous and a consensus or a proven, clearly superior strategy is still not apparent [11, 12]. The published studies differ much in their results and lead to contradictory recommendations. This shows that the correlations are not yet clear and suggests that the factors influencing the infection rates are diverse and that the type and length of antibiotic prophylaxis may be perhaps less decisive than assumed. Thus, despite the numerous published studies, further research on this topic is still important. The wide range of results in the rate of SSI in orthognathic surgery and the ongoing controversial debate on the necessity of a prolonged antibiotic prophylaxis in order to reduce SSI in highly elective procedures encouraged us to conduct this study. Another point were the possible adverse effects of a prolonged application of antibiotics on the individual and on society.

A large double-blind study with 14 participating centers, initiated by Ristow et al. [13], in 2021, is intended to bring more clarity. In preparation for participation in this trial.

The purpose of the present study was therefore to add further clinical results to the existing data in literature. We aimed to retrospectively determine the SSI rates for these interventions in our series and to discuss antibiotic prophylaxis, but also to detect other possible influencing factors in addition to the antibiotic prophylaxis regime. The null hypothesis was, that a prolonged antibiotic prophylaxis regime has no influence on the rate of SSI in orthognathic surgery.

## Material and methods

### Study design and data collection

Our electronic database was searched for patients with dentofacial anomalies and malocclusions who underwent orthognathic surgery in our department between 2010 and 2020. We only included patients with complete clinical and radiological documentation as well as patients who received one-jaw (bilateral sagittal split osteotomy (BSSO) or Le Fort I-osteotomy) or bimaxillary, two-jaw surgery (BSSO and Le Fort I). The exclusion criteria were patients with incomplete documentation as well as patients who received surgically assisted rapid palatal expansion (SARPE), Le Fort II or Le Fort III osteotomy, segmental mandibular or maxillary osteotomy or genioplasty. All procedures were performed after proper orthodontic preparation. Surgeries were conducted by 3 surgeons during the observation period. Surgeon 2 and 3 were scholars of the first one using the identical surgical technique. For maxillary movement, Le Fort I osteotomy was performed in all included patients as previously described [14, 15]. In the mandible, bilateral sagittal split osteotomy (BSSO) was performed according to Obwegeser [16] and its later modifications [17, 18]. In all cases, titanium miniplate osteosynthesis was performed for internal fixation. There was no strict scientific follow-up protocol. Patients received regular surgical follow-up postoperatively, depending on clinical necessity, until the start of orthodontic postoperative treatment. Thereafter, regular surgical follow-up usually ended with the removal of miniplates which was scheduled after 6–9 months. The length of follow-up after the second surgical intervention was again made dependent on individual clinical requirements. Afterwards, a revisit only took place if necessary.

We retrospectively analysed clinical records, anaesthesia protocols and operative charts. Clinical characteristics of patients, pre-existing condition, age and previous operations were documented. For analysis patients were not age-matched. Also, no predictors or effect modifiers were previously defined. Details about surgical management such as treatment approaches (one- vs. two-jaw surgery), displacement distances and operating time were collected. Perioperative infection prophylaxis (e.g., antibiotic regime, type of drainage,) as well as surgical site infections (SSIs) and their management were assessed. Any patient with prophylactic or intraoperative antibiotic were enrolled. SSIs were defined by standardised surveillance criteria [19]. SSIs occurring within one year postoperatively were considered according to these criteria for inserted implants. In addition, infections were clinically divided into uncomplicated wound infections and complicated postoperative infections. Uncomplicated wound infections were classified as infections treated locally with cooling and irrigation of the surgical wound with or without additional antibiotic therapy. In contrast patients with complicated postoperative infections required surgical wound revision with intraoral incision and drainage as well as additional antibiotic therapy.

Besides the applied antibiotic regime, also other possible influencing factors and confounders were assessed and analysed including the demographic factors (i.e. age at surgery and gender) and associated craniofacial malformations as well as smoking, site of infection, type and duration of surgery, displacement distances, type of drainage.

This study was carried out in accordance with the ethical standards and recommendations of the institutional ethics committee (472/2019BO2). The STROBE reporting guidelines were followed.

### Data analysis

Patient data were collected and pseudonymised from our electronic database and clinical records. Statistical evaluation and descriptive statistics were performed using SPSS Version 24.0 (IBM, Armonk, NY). Shapiro–Wilk *test was performed for normally distributed data.* For statistical analyses non-parametric Mann–Whitney-U-tests were applied. Pearson’s chi-squared test was carried out to compare categorical parameters. Statistical significance was considered if *P* < 0.05. Numerical data are presented as numbers and percentages, mean and standard deviation, or median and interquartile range. On purpose we did not perform a sample size-calculation before conducting the study as Ristow et al. have already published such one. They calculated a necessary sample size of 1400 patients (13) in order to answer the question whether a prolonged antibiotic prophylaxis regime reduces the risk of SSI.

## Results

### Patient characteristics (Table 1)

**Table 1:** Patient characteristics

Patients [n (%)]		291	(100)
Age [years (± SD)]		25.5	(± 8.5)
Gender [n (%)]	Female	165	(56.7)
	Male	126	(43.3)
Craniofacial anomalies [n (%)]		54	(18.6)
Previous surgery [n (%)]		75	(25.7)
Orthognathic surgery [n (%)]	Bimax	158	(54.3)
	BSSO	80	(27.5)
	Le Fort I	53	(18.2)
Distances [mm (± SD)]	maxillary advancement	3.4	(± 2.1)
	mandibular advancement	4.6	(± 2.3)
	mandibular setback	-4.3	(± 2.1)
Surgery duration [minutes (± SD)]		249	(± 97.4)

*Bimax* bimaxillary two-jaw surgery, *BSSO* bilateral sagittal split osteotomy

In the initial enrolment 405 patients, operated between 2010 and 2020, were assessed for eligibility; 114 of these (28%) met the exclusion criteria. We thus included 291 patients (43.3% male, 56.7% female) in this retrospective observational study. The mean age at surgery was 25.5 ± 8.5 years. Fifty-four patients (18.6%) were diagnosed with a craniofacial malformation, the majority suffering from cleft lip and/or palate (CL/P) (*n* = 42), followed by rare conditions such as hemifacial microsomia (*n* = 5), complex craniofacial deformities (*n* = 3), hemimandibular hyperplasia (*n* = 2), Treacher Collins syndrome (*n* = 1) and Moebius syndrome (*n* = 1). Relevant previous surgeries were documented in about one quarter of included patients (*n* = 75). Most of these underwent cleft surgery (*n* = 42, 14.4%) or received surgically assisted rapid palatal expansion (SARPE) before orthognathic surgery (*n* = 26, 8.9%). A few cases had previously received distraction osteogenesis of the mandible (MDO) due to hemifacial microsomia (*n* = 3), complex fracture treatment (*n* = 3) or high condylectomy due to hemimandibular hyperplasia (*n* = 1).

In total 291 orthognathic procedures were analysed. In the majority of cases bimaxillary, two-jaw surgery was performed (*n* = 158, 54.3%), whereas one-jaw surgery of the mandible (BSSO) was performed in 80 patients (27.5%) and one-jaw maxillary osteotomy (Le Fort I osteotomy) in 53 patients (18.2%). The overall maxillary advancement was 3.4 (± 2.1) mm. The mean displacement in the mandible was 4.5 (± 2.2) mm, the mean mandibular advancement was 4.6 (± 2.3) mm and the mean mandibular setback was -4.3 (± 2.1) mm. The overall mean duration of surgery was 249 (± 97.4) min: 318 (± 71.2) min for two-jaw surgery, 172 (± 39.4) min for one-jaw BSSO, and 158 (± 52.0) min for one-jaw Le Fort I osteotomy. The mean follow-up period was 11.4 (± 6.6) months.

### Perioperative infection prophylaxis (Table 2)

**Table 2:** Perioperative infection prophylaxis

Antibiotic regime [n(%)]	single-dose	267	(91.8)
	prolonged	24	(8.2)
Antibiotics [n(%)]	cefuroxime	188	(64.6)
	penicillin G	87	(29.9)
	clindamycin	16	(5.5)
Type of drainage mandible (*n* = 238) [n(%)]	suction drainage	218	(91.6)
	capillary drains	15	(6.3)
	no drainage	5	(2.1)
Intraoperative glucocorticoid [n(%)]		236	(81.1)

*Bimax* bimaxillary two-jaw surgery, *BSSO* bilateral sagittal split osteotomy

Ninety-two percent of patients (*n* = 267) received intraoperative single-dose antibiotic prophylaxis, whereas 8% (*n* = 24) received prophylactic prolonged postoperative antibiotics for 5 days. The statistical analysis revealed no significant difference in the occurrence of SSI between these two groups (*P* > 0.05). During the study period, there was a switch from penicillin G (*n* = 87, 29.9%) to the now commonly used cefuroxime (*n* = 188, 64.6%). In case of documented allergy or hypersensitivity against penicillin derivatives, clindamycin was used (*n* = 16, 5.5%). The statistical analysis showed no significant influence of the selected antibiotic on the occurrence of SSI (*P* > 0.05). After BSSO, patients usually received suction drainage in the mandible (*n* = 218/238, 91.6%). In rare cases patients received silicone capillary drains (*n* = 15/238, 6.3%) or no drainage (*n* = 5/238, 2.1%). Wound drainage in the maxilla was not performed in any patient. Intraoperative glucocorticoid administration for reducing postoperative swelling was documented in 81% of patients. In addition, all patients received controlled postoperative cooling using a Hilotherm® face mask (Hilotherm GmbH, Argenbühl-Eisenharz, Germany).

### Surgical site infections (Table 3)

**Table 3:** Surgical site infections

ID	Sex	Age at surgery	Diseases	Previous surgery	Orthodontic surgery	Infection site	OAI	Classification of infection	Days until infection
1	M	49			Bimax	mand	n	UC	5
2	F	41			BSSO	mand	n	UC	9
3	M	24			Bimax	mand	n	UC	2
4	M	27		SARPE	Bimax	mand	y	UC	78
5	M	27			Bimax	mand	n	UC	7
6	F	30			BSSO	mand	n	UC	6
7	F	28			BSSO	mand	n	UC	9
8	F	28		SARPE	BSSO	mand	n	CI	14
9	F	18			BSSO	mand	n	UC	2
10	M	37			BSSO	mand	n	CI	39
11	F	23		SARPE	Bimax	mand	y	CI	78
12	M	21			Bimax	max	n	UC	9
13	F	44			BSSO	mand	n	CI	49
14	F	20			Bimax	mand	y	CI	72
15	F	20			Le Fort I	max	y	CI	37
16	F	17			Bimax	mand	n	UC	13
17	F	19			Bimax	mand	n	UC	20
18	F	27			BSSO	mand	n	UC	8
19	M	21	CP	cleft surgery + SARPE	BSSO	mand	y	CI	128
20	M	19			Bimax	mand	n	CI	42
21	M	32		SARPE	Bimax	max	y	CI	13
22	F	16			Bimax	mand	n	UC	12
23	F	34		SARPE	BSSO	mand	y	CI	82
24	F	21			Bimax	max	y	UC	20
25	M	19	BCLP	cleft surgery	Bimax	mand	n	UC	29
26	M	28			Bimax	mand	n	UC	6
27	M	18	BCLP	cleft surgery	Bimax	mand	y	CI	74
28	M	18	UCL	cleft surgery	Bimax	max	n	UC	78
29	F	28			Bimax	mand	n	CI	27
30	F	22			Bimax	mand	y	CI	194
31	F	32			BSSO	mand	n	UC	6
32	F	25		SARPE	BSSO	mand	n	UC	18
33	M	27	HH	HC	Bimax	mand	n	UC	13
34	F	19			Bimax	mand	n	UC	6
35	M	21	HM	MDO	Bimax	mand	y	CI	52
36	F	22			Bimax	mand	y	UC	8

*M* male, *F* female, *OAI* osteosynthesis-associated infection, *BCLP* bilateral cleft lip and palate, *UCL* unilateral cleft lip, *CP* cleft palate, *HH* hemimandibular hyperplasia, *HM* hemifacial microsomia, *SARPE* surgically assisted rapid palatal expansion, *HC* high condylectomy, *MDO* mandibular distraction osteogenesis, *Bimax* bimaxillary two-jaw surgery, *BSSO* bilateral sagittal split osteotomy, *max* maxilla, *mand* mandible, *UC* uncomplicated infection, *CI* complicated infection

Surgical site infections occurred in 12.4% (*n* = 36) of patients. The majority of infections were located in the mandible (*n* = 31/36). Five patients (*n* = 5/36) presented a postoperative SSI in the maxilla. Furthermore, 23 SSIs occurred after bimaxillary two-jaw surgery, 12 after one-jaw BSSO and one after one-jaw Le Fort I osteotomy. Twelve patients were diagnosed with an osteosynthesis-associated infection (OAI) (*n* = 9 mandible, *n* = 3 maxilla). The median postoperative interval until infection was 16.0 days (2–194, IQR 43), whereas OAI occurred significantly later (73.0 (IQR 57) vs.10.5 (IQR 19), *P* = 0.000). Infections were classified clinically into uncomplicated wound infections and complicated postoperative infections. Twenty-two patients (7.6%) had uncomplicated wound infections. Out of these 4 patients were treated locally with cooling and irrigation of the surgical wound. Another 18 patients required antibiotic therapy in addition to local therapy. In contrast, 14 patients (4.8%) developed complicated postoperative SSIs after orthognathic surgery. They required surgical wound revision with intraoral incision and drainage as well as additional antibiotic therapy. Table 3 displays the clinical details of the patients who presented with postoperative infection. None of the SSIs had a significant effect on the final surgical outcome.

There was only one complication during surgery in the SSI group. One 34-year-old female patient (ID 23) had an intraoperative bad split during BSSO in bimaxillary surgery. However, we were able to continue surgery. This patient developed an osteosynthesis-associated infection (OAI) and was treated with incision and drainage as well as antibiotic therapy and early removal of miniplates (also see Table 3). None of the patients required a blood transfusion. There was also no prolonged stay due to SSI. The median (min–max) stay in the non-affected group was 6 days (3–12 days) and 6 days (3–9 days) in the SSI group.

Further statistical analyses were performed to investigate multiple co-factors. There was a higher percentage of smokers in the infection group (χ^2^(1) = 4.4, *P* = 0.036). Fourteen out of 36 patients in the infection group were smokers (38.9% vs. 22.7%). In addition, the prevalence of SARPE (*n* = 7) was significantly higher (χ^2^(1) = 5.6, *P* = 0.018) in the infection group, whereas no significant difference in the prevalence of craniofacial malformations was detected between groups (χ^2^(1) = 0.1, *P* = 0.755).

Analyses revealed a significant association (χ^2^(2) = 6.58, *P* = 0.037) between postoperative infections and the type of surgery (i.e., BSSO (*n* = 12/80), Le Fort I (*n* = 1/53) and bimaxillary surgery (*n* = 23/158)). There was also a significant association (χ^2^(2) = 12.5, *P* = 0.002) between postoperative infection and type of drainage (i.e., suction drainage (*n* = 30/218), capillary drains (*n* = 5/15), no drainage (*n* = 1/58)). Furthermore, no significant relationships were observed between postoperative infections and various co-factors (i.e., age at surgery, gender, associated craniofacial malformations, surgery duration, displacement distances, mandibular setback vs. advancement) (*P* > 0.05).

## Discussion

Surgical site infections are a relevant complication following orthognathic surgery and are associated with further burden both for the patient and the medical care system.

The current study revealed an overall infection rate of 12.4% within 1 year following orthognathic surgery. This is well within the infection rate of 10–15% expected by Peterson et al. [1] for class II procedures as well as in the lower half of the range (1.4–33.4%) specifically reported for orthognathic procedures [2, 3]. With regard to our regime of administered antibiotics, this rate is below the data given by some authors for a single-dose regimen [2, 8, 20]. On the other hand, the literature also shows rates well below 12.4% for a prolonged regimen [7, 10, 21]. No clear superiority of the single-dose regimen can be proven with our data and this study design. But as most patients (91.8%, *n* = 267) received a single-dose of antibiotic prophylaxis, it can be concluded that abstaining from prolonged prophylaxis at least does not lead to an exceptionally high infection rate. A statistical correlation between antibiotic regime and infection rate could not be proven on the basis of these data. Insofar the null hypothesis could be accepted with the limitation of a small number of patients with a prolonged antibiotic prophylaxis. Furthermore, none of the SSIs negatively affected the surgical outcome. In addition to the infection rate, however, the risks of prolonged prophylaxis should also be taken into account, such as the dramatic development of resistance and the associated increase in the number of deaths worldwide predicted by the WHO [4] or adverse side effects associated with the administration of antibiotics. Davis et al. [22] indicated a rate of 4.2% for adverse effects of antibiotic administration. In addition, the majority of reported SSIs were of minor severity. Furthermore, it is unlikely that late osteosynthesis-associated infections with a median postoperative interval until infection of 73.0 days could be avoided by prolonged antibiotic administration immediately postoperatively. All these aspects provide further justification for preferring the single-dose to a prolonged regimen. This is in line with some comparative studies, e.g. Gaal et al. [23] and Ghantous et al. [5] found no advantage of prolonged prophylaxis.

There was also no correlation with many other co-factors such as age, gender, duration of surgery and displacement distance or type. Concerning gender and age the data confirm the findings of Posnick et al. [10] but are contrary to Barrier et al.[24] with regard to the duration of surgery. Especially for the large anterior displacement distances the result was surprising. We would have assumed, that less bone contact might induce an increased risk of SSI. In contrast and as expected, smoking was found to be a relevant factor, thus confirming the findings of Kuhlefelt et al. [25], but contrary to the data of Davis et al. [22]. Further studies, which should record the duration and quantity of tobacco consumption both pre- and postoperatively more precisely and evaluate it in a differentiated manner, are needed to clarify this matter.

The outcome of the evaluation of the influence of pre-existing craniofacial malformations on the infection rate was surprising. The study cohort included a high percentage (*n* = 54, 18.6%) of patients with concomitant craniofacial malformations. Most of them had multiple previous surgical interventions, which were expected to negatively influence the infection rate and represent a risk factor. This assumption was refuted by the data analysis. While the statistical evaluation revealed no correlation between SSIs and craniofacial malformations, a significant association was detected between infections and type of surgery (χ^2^(2) = 6.58, *P* = 0.037). The majority of infections occurred in the mandible (*n* = 31/36). This is consistent with the data of other authors [22, 25, 26]. This may be due to limited blood supply of the mandible compared to the maxilla. In the cohort of patients with craniofacial malformations, clefts were predominant (*n* = 42/54) and most of them required only a single-jaw surgery with maxillary advancement to correct the cleft-associated maxillary retrognathia. Davis et al. [22], among others, reported a significantly lower infection rate with a single Le Fort I osteotomy. This might explain why craniofacial anomalies were not statistically associated with the occurrence of SSI. However, 4 out of 42 cleft patients had an SSI after orthognathic surgery. All 4 had received a bimaxillary procedure and 3 of them developed an infection in the mandible. No cleft patient with single maxillary advancement had a postoperative infection. The location and type of surgery is more relevant for the infection risk than associated craniofacial malformations. Interestingly, however, the proportion of patients with previous SARPE was also significantly higher in the infection group (*n* = 7), but the infection was again mostly located in the mandible (*n* = 6) and only one case had a wound infection in the maxilla. A plausible explanation for this correlation cannot be given. It should be confirmed or refuted by much larger studies.

Another perioperative measure had a significant impact on the postoperative infection rate. Analysis revealed an advantage of suction drains compared to capillary drains. This is consistent with the basic idea that the smaller the haematoma, the lower the risk of infection. For better patient comfort, the well-established practice of suction drains was changed and only capillary drains were inserted after BSSO. However, after only a short time, a significant increase in infection rates was observed in these patients, so this approach was quickly abandoned. Looking at the literature the present results contradict the findings of Spaey et al. [26] and Kuhlefeldt et al. [25] who reported increased infection rates when using drains. Further studies with large case numbers would be useful in this regard.

Another issue to discuss are the surgery room conditions. Laminar air flow is generally controversially discussed. But Barbadoro et al.[27] found out that there is a benefit of laminar air flow for clean as well as for clean-contaminated surgery. Orthognathic surgery belongs to clean- contaminated surgery. As all operating rooms of our hospital are equipped with laminar air flow, all procedures took place under this condition.

The above observations illustrate the limitations of the study.. The retrospective nature of the study is accompanied by a number of limitations, not all of which can be eliminated. Although the STROBE checklist served as a guideline, not all items could be adequately addressed due to the retrospective approach. Non-randomized and retrospective observational studies in particular are subject to the dangers of bias in a variety of ways and at all levels (patient selection, collection, evaluation and interpretation of the data).

For example, the patient clientele is not balanced, neither to gender, nor to age, but all treated orthognathic surgery cases were initially considered and checked for the inclusion and exclusion criteria. In addition, there are neither uniform follow-up intervals, nor is the documentation of the data uniform. Therefore, a selection bias as well as an information bias cannot be completely excluded. Possible confounding factors may be e.g. concomitant diseases. Craniofacial abnormalities have been explicitly mentioned, but reliable information on other potentially influencing pre-existing conditions is not consistently available. However, the latter is less relevant in its impact as a potential confounder factor, since most patients for elective orthognathic surgery are young, healthy people.

Nevertheless, we consider the information gained from this study to be valuable, especially in addition to existing literature. All retrospective observational studies are afflicted with similar problems. Pointing out and keeping in mind possible biases is probably the best way to address these dangers as they cannot be eliminated. Thus, this study, like most others, cannot clearly determine the best antibiotic prophylactic regime in orthognathic surgery. Unfortunately, very large numbers of cases are needed to provide solid statistical evidence of the superiority of a specific prophylaxis regime. Insofar the number of cases is another limitation of our and most other studies. More than 1400 cases are necessary for a statistically supported statement on this topic [13]. This is hardly feasible for a single centre in an acceptable time frame. Accordingly, the literature usually contains either prospective, randomised studies with quite small numbers [6, 7, 28–30] or retrospective studies with larger numbers of cases [2, 3, 9, 20, 22, 23, 31]. Both approaches clearly minimise the significance. This aspect has been repeatedly criticised in several published reviews and meta-analyses [11, 12, 21]. Due to these difficulties, the controversial debate about the optimal antibiotic prophylaxis regimen is still ongoing and high-quality randomised studies investigating this issue are needed. With the intention of overcoming these limitations and achieving case numbers of over 1400, Ristow et al. [13] have initiated a large multicentre randomised double-blind clinical trial also involving our centre.

In summary, the pure single-dose antibiotic protocol for the prophylaxis of SSI in orthognathic surgery led in our series to SSI rates lying within the superior range reported in literature. Other factors as the type of drainage, site of surgery or smoking are statistically significant. According to our analysis, craniofacial anomalies do not represent a risk factor in this regard. The present data are a valuable addition to the existing literature and also reveal aspects that are worth further, more detailed investigation.

## Data Availability

The datasets generated and/or analysed during the current study are available from the corresponding author on reasonable request.

## Abbreviations

BSSO: Bilateral sagittal split osteotomy
CL/P: Cleft Lip and/or Palate
MDO: Mandibular distraction osteogenesis
OAI: Osteosynthesis-associated infection
SARPE: Surgically assisted rapid palatal expansion
SSI: Surgical site infection
WHO: World health organisation
